# WHO DIES IN A RURAL NURSING HOME WITH AND WITHOUT FAMILY? EVIDENCE FROM THE UTAH POPULATION DATABASE

**DOI:** 10.1093/geroni/igad104.1710

**Published:** 2023-12-21

**Authors:** Rebecca Goodwin, Katherine Ornstein, Eli Iacob, Michael Hollingshaus, Ken Smith, Djin Tay, Rebecca Utz, Caroline Stephens

**Affiliations:** National Library of Medicine, NIH & University of Utah College of Nursing, Salt Lake City, Utah, United States; Johns Hopkins University, Baltimore, Maryland, United States; University of Utah, Salt Lake City, Utah, United States; University of Utah, Salt Lake City, Utah, United States; University of Utah, Salt Lake City, Utah, United States; University of Utah, Salt Lake City, Utah, United States; University of Utah, Salt Lake City, Utah, United States; University of Utah, Salt Lake City, Utah, United States

## Abstract

Rural nursing homes (NHs) are an increasingly important focus for policy makers given the lack of available post-acute and long-term care services in these areas. Families often play a critical role in filling this void in services and supporting NH residents at the end of life (EOL). No population-based studies to date, however, have documented the characteristics of families of persons who die in rural NHs. This study aimed to describe the size and composition of first-degree families (FDF) of Utah NH residents who died in rural/frontier NHs 1998-2016 (n=10,567). Using the Utah Population Caregiving Database, we linked rural/frontier NH decedents to their FDF (n=31,118; children=53.7%; siblings=33.8%; spouses=11.3%; parents=1.1%) and compared characteristics of those with and without FDF. Compared to rural/frontier NH decedents with FDF (77.5%), those without (22.5%) were more likely to be older (median 85.7 vs 84.8), female (61.3% vs 56.8%), Hispanic or Non-White Non-Hispanic (6.8% vs 3.7%), and less educated (<9th grade; 45.9% vs 37.4%), all p<0.001. There were no differences in diagnosis of dementia, Charlson Comorbidity Index, or hospitalizations in the last 6 months of life. Persons who live and die in rural NHs without FDF may be in double jeopardy, if you will, given the lack of family support and few to no options for care. Understanding the impact of family, or lack thereof, on rural NH resident EOL care trajectories is an important next step.

